# Time Intervals Under the Lens at Sweden’s First Diagnostic Center for Primary Care Patients With Nonspecific Symptoms of Cancer. A Comparison With Matched Control Patients

**DOI:** 10.3389/fonc.2020.561379

**Published:** 2020-11-30

**Authors:** Jan Sundquist, Karolina Palmér, Stefan Rydén, Charlotta Sävblom, Jianguang Ji, Emelie Stenman

**Affiliations:** ^1^ Center for Primary Health Care Research, Skåne Regional Council, Lund University, Malmö, Sweden; ^2^ Department of Family Medicine and Community Health, Department of Population Health Science and Policy, Icahn School of Medicine at Mount Sinai, New York, NY, United States; ^3^ Center for Community-based Healthcare Research and Education (CoHRE), Department of Functional Pathology, Shimane University, Shimane, Japan; ^4^ Regional Cancer Centre South, Skåne Regional Council, Kristianstad, Sweden; ^5^ Regional Cancer Centre Stockholm Gotland, Stockholm Regional Council, Stockholm, Sweden

**Keywords:** cancer, nonspecific symptoms, time intervals, diagnostic center, primary care, diagnostic interval

## Abstract

**Introduction:**

Fast-track referral pathways for patients with nonspecific, serious symptoms have been implemented in several countries. Our objective was to analyze time intervals in the diagnostic routes of patients diagnosed with cancer at Sweden’s first Diagnostic Center (DC) for nonspecific symptoms and compare with time intervals of matched control patients.

**Methods:**

Adult patients with nonspecific symptoms that could not be explained by an initial investigation in primary care were eligible for referral to the DC. Patients diagnosed with cancer were matched with patients at another hospital within the same healthcare organization. We aimed for two control patients per DC-patient and matched on tumor type, age and sex. Five time intervals were compared: 1) patient interval (first symptom—primary care contact), 2) primary care interval (first visit—referral to the DC/secondary care), 3) diagnostic interval (first visit—cancer diagnosis), 4) information interval (cancer diagnosis—patient informed) and 5) treatment interval (cancer diagnosis—treatment start). Comparisons between groups and matched cohort analyses were made.

**Results:**

Sixty-four patients (22.1%) were diagnosed with cancer at the DC, of which eight were not matchable. Forty-two patients were matched with two controls and 14 were matched with one control. There were no significant differences in patient-, primary care-, or diagnostic intervals between the groups. The information interval was shorter at the DC compared to the control group (difference between matched pairs 7 days, p = 0.001) and the treatment interval was also shorter at the DC with significant differences in the matched analysis (difference between matched pairs 13 days, p = 0.049). The findings remained the same in four sensitivity analyses, made to compensate for differences between the groups.

**Conclusions:**

Up to diagnosis, we could not detect significant differences in time intervals between the DC and the control group. However, the shorter information and treatment intervals at the DC should be advantageous for these patients who will get timely access to treatment or palliative care. Due to limitations regarding comparability between the groups, the results must be interpreted with caution and further research is warranted.

**Trial registration:**

ClinicalTrials.gov-ID: NCT01709539. Registration-date: October 18, 2012.

## Introduction

Fast-track referral pathways for patients with nonspecific symptoms that may indicate cancer are becoming increasingly widespread. The concept “Nonspecific symptoms and signs of cancer patient pathway” (NSSC-CPP) was invented in Denmark (1, 2) and is currently part of the national cancer strategies in all the Scandinavian countries (2–4). In the UK, a new Suspected CANcer (SCAN) pathway for patients with “low-risk but not no-risk” is currently being evaluated (5). Sweden has a relatively high cancer survival rate (6, 7). Nevertheless, the healthcare system has waiting time problems compared with European counterparts; owing to clinically excellent healthcare services, Sweden is one of eight out of 35 countries scoring > 800 out of 1000 in the Euro Health Consumer Index (in which 1000 indicates highest possible standards), but only two countries scored lower on the accessibility/waiting time indicator (8).

Whether there is an association between time to cancer diagnosis and outcome has been debated. Some studies have failed to demonstrate such an association while other studies have shown a negative association with worse outcome for shorter diagnostic or treatment intervals, or a u-shaped association with poorer outcome for the shortest and longest diagnostic intervals (9, 10). However, we know that a more advanced tumor stage is associated with worse prognosis (11, 12) and several studies have shown favorable clinical outcome with shorter diagnostic intervals (9, 10, 13–16). Thus, aiming for timely cancer diagnoses is highly motivated. For some cancer diagnoses, patients with vague symptoms have had longer diagnostic intervals compared to patients with alarm symptoms (16). Thus, when Denmark presented its NSSC-CPP concept, researchers at the Regional Cancer Center in southern Sweden became interested and started a project with a similar pathway for patients in primary care that showed one or more of a number of pre-specified nonspecific symptoms. This resulted in Sweden’s first diagnostic center (DC), which was established at the central hospital of Kristianstad. The project involved 42 primary care centers within a catchment area of 220 000 inhabitants. If initial investigations and tests in primary care did not explain the patients’ symptoms, the patients were eligible for referral to the DC. At the DC, which has direct access to imaging facilities (computed tomography, positron emission tomography, ultrasound, and magnetic resonance imaging) and consultants specializing in different medical areas, a comprehensive investigation was performed until diagnosis was identified or ruled out (3).

**Figure 1:**
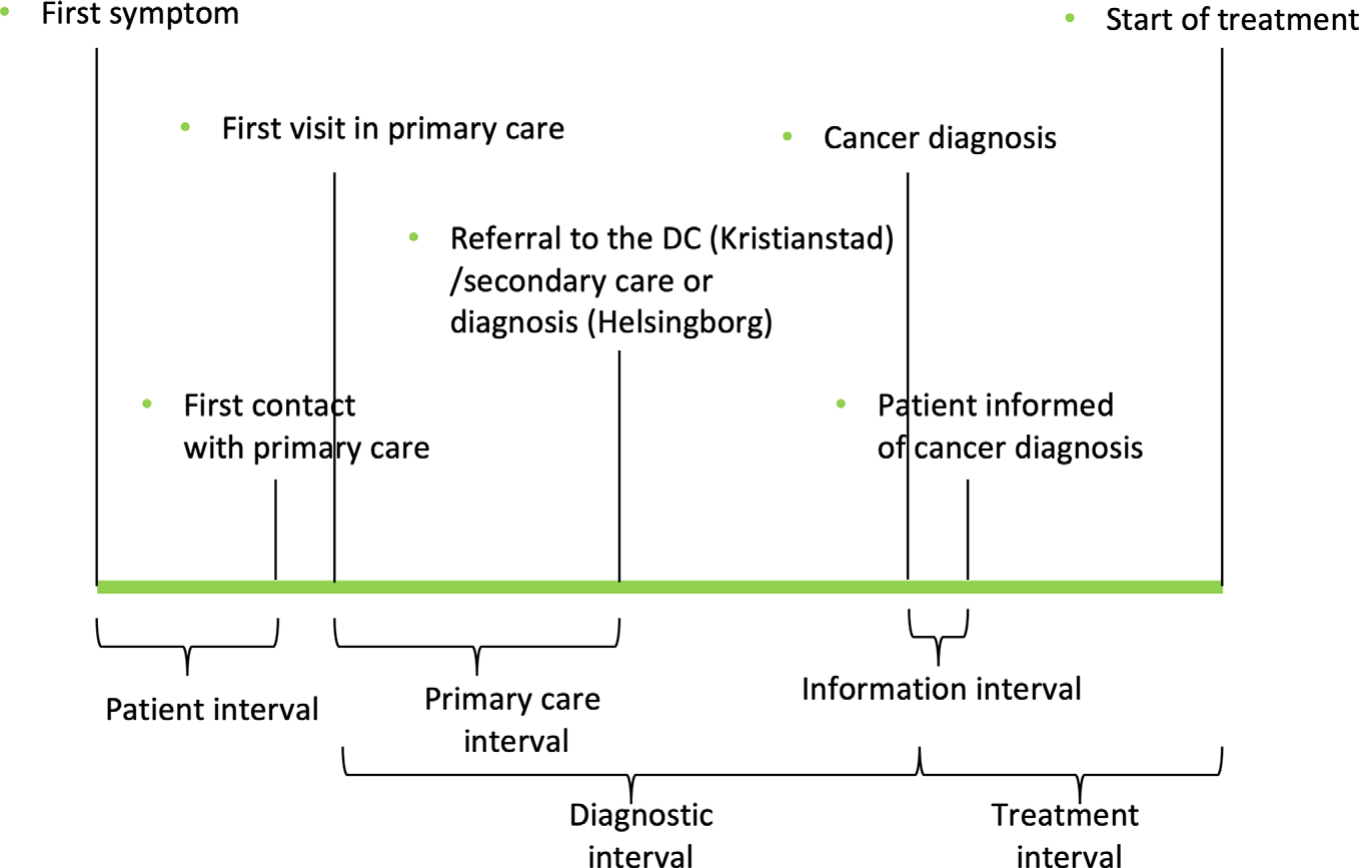
Illustration of the different time intervals in the study.

The main goals with the Swedish DC were to reduce the time to cancer diagnosis for primary care patients with nonspecific symptoms and to make the diagnostic process more effective and standardized. This could, however, not be evaluated in a randomized controlled trial since the concept was implemented in the whole catchment area simultaneously and randomizing the patients was considered unethical. Diagnostic time intervals of Danish NSSC-CPP’s have been presented (17, 18) but, to our knowledge, it has not been examined in controlled studies. Thus, to put time intervals into context and get a rough estimation of whether the DC could reduce the time to diagnosis, we decided to compare patients that were diagnosed with cancer at the DC with matched control patients at another hospital of a similar size within the same healthcare region, but with a different geographical catchment area, the Helsingborg hospital. The specific aim of the present study was to compare five different time intervals between the DC and the matched control group; 1) the patient interval, 2) the primary care interval, 3) the diagnostic interval, 4) the information interval and 5) the treatment interval (explained in Data collection and **Figure 1**).

## Materials and Methods

We compared patients that were diagnosed with cancer at the DC, at the central hospital of Kristianstad, (exposed group) with patients that were diagnosed with cancer at Helsingborg hospital during the same time period (control group). Both hospitals are emergency hospitals within the same healthcare organization with similar guidelines and routines, but in different geographical areas, in Sweden’s southernmost county, Skåne (Scania; 1.3 million residents).The study was approved by the Regional Ethics Committee in Lund, Sweden, registration number 2012/449.

### Exposed Group: Patients That Were Diagnosed With Cancer at the Diagnostic Center

The diagnostic process and setting of the first Swedish DC has been described in detail previously (3). The DC was established in October 2012 as a separate, outpatient unit within the department of internal medicine in close collaboration with the radiology department. The overall evaluation of the DC, of which this study was one part, was performed as a prospective cohort study. Patients that were referred to the DC from primary care during the center’s first 3 years were consecutively invited to participate in the study in connection with their first visit at the DC. The only exception was for those who were unable to provide informed consent based on oral and written study information in Swedish. Primary care physicians were invited to refer patients aged 18 years or older. Inclusion criteria for referral to the DC included cancer suspicion and one or more of the following symptoms (adapted from the Danish model): 1) fatigue, 2) weight loss more than 5 kg, 3) pain/joint pain, 4) prolonged fever, 5) abnormal test results, or 6) suspected metastasis of unknown origin (19). The diagnostic workup in primary care included a clinical examination, two standardized sets of laboratory tests, chest X-ray and abdominal ultrasound. If no explanation for the symptoms was found, the patients were eligible for referral to the DC. Referral to the DC was not encouraged for patients with alarm symptoms or when a cancer diagnosis was confirmed in primary care. These patients should be referred directly to the appropriate secondary care unit.

In conjunction with referral to the DC, another comprehensive panel of laboratory tests (the DC-package) was taken. At the DC, a thorough physical examination was performed and appropriate further investigations, including consultations with other specialists, were done until a diagnosis was identified or disease could be ruled out. In the present study, only patients that were diagnosed with cancer at the DC were included in the analyses. Recruitment of study participants started in October 2012 and was planned to continue until 60 patients were diagnosed with cancer at the DC, which occurred in September 2015. This number emanated from an expected 20% cancer prevalence (based on preliminary data from Denmark at the time of the project) and the expected need for 300 patients in order to ensure proper validation of the laboratory tests in the DC-package.

### Control Group: Matched Patients Diagnosed With Cancer at Helsingborg Hospital

For every patient diagnosed with cancer at the DC, we aimed for two matched control patients diagnosed with cancer at Helsingborg hospital. We identified 2,221 patients diagnosed with cancer in the catchment area of Helsingborg hospital from 2013 through 2015 in the Swedish Cancer Register, which comprises all newly detected cancer cases in Sweden. Of these, 428 were considered possible to match with DC-patients based on cancer type. The rest had completely different cancer diagnoses that were not considered comparable. Contact nurses at Helsingborg hospital checked in the medical records of the possible control patients if they were eligible for the study (i.e. the diagnostic process started in primary care and the patient was still alive, thus could consent to take part and provide information about time for first symptom). Eligible patients were contacted at the clinic or by telephone. Patients who agreed to participate were given or sent a study information leaflet and were invited to respond with a written informed consent form along with a short questionnaire including time from first symptom to first contact with primary care. In the end, 113 eligible patients (26%) consented to take part in the study. One control patient per DC-patient was selected based on best possible match of three criteria (in priority order): 1) tumor type (ICD-code); 2) age (± 5 years); and 3) sex. Of the remaining control patients, a second best matched patient was then selected, if possible. Of the total 98 matched pairs, 24% were matched to the exact same tumor type (ICD-code), 36% were matched to similar tumor types (for example ICD-code C343 were matched to C349). Thirty-seven percent were matched to alternative cancer ICD-codes deemed by a physician to require approximately similar diagnostic intervals. Three percent were considered “worst match” and included, for instance, C829 matched to C110. In a sensitivity analysis, we excluded the 40% of the pairs that were not matched on exact or similar tumor types.

### Data Collection

Data on basic patient characteristics, time points during the cancer investigations, symptoms, diagnoses and comorbidity (defined by ICD-codes) were collected from examinations, referrals and the medical records in case report forms by the physician and nurse at the DC and from medical records by study nurses at Helsingborg hospital. The data was monitored and validated by a research nurse using original data from the medical records.

The terms “patient interval”, “primary care interval”, “diagnostic interval” and “treatment interval” were adapted from the Aarhus statement (20). The patient intervals were self-reported by all patients with a multiple choice question about the time (in categories) from first symptom to first contact with primary care. Dates for the other intervals were collected from case report forms and medical records. The primary care interval was defined as the time between first doctor’s appointment in primary care to referral to the DC/secondary care (or in some cases diagnosis; in the exposed group all patients were referred to the DC, whereas in the control group, 17 (17%) patients received their diagnosis in primary care, thus the primary care interval was equal to the diagnostic interval). The diagnostic interval was defined as time for first doctor’s appointment in primary care to date of cancer diagnosis first noted in the medical records. If a patient got more than one cancer diagnosis during the same investigation, the first date was chosen. Information interval was defined as the time from the first note of cancer diagnosis in the medical records to the time when the patient was informed about the diagnosis. Treatment interval was defined as the time from cancer diagnosis to start of treatment (**Figure 1**).

The following symptoms were considered focal alarm symptoms, based on Swedish national and regional recommendations and a study about warning signs of cancer (21): fecal blood, hemoptysis, hematuria, postmenopausal bleeding, lump, irregular prostate shape, dysphagia, non-healing lesion, pigmented skin lesion/mole, and recurrent laryngeal nerve paralysis. Infiltrating tumors were defined as solid tumors with potential to spread based on TNM-staging. These tumors were identified in the Swedish Cancer Register by an evaluation of the combined TNM-codes for each patient.

### Statistical Analysis

Age was presented as median and interquartile range (percentiles 25–75; IQR). Sex, cancer diagnoses, previous diagnoses, symptoms and alarm symptoms were presented as numbers and percentages. Data concerning survival rates for patients with cancer were obtained from the Swedish Cancer Register and presented as 3-year survival rate with 95% confidence intervals (CI).

We examined the five different time intervals separately at the DC and in Helsingborg and compared them by using nonparametric tests. The patient intervals were presented with numbers and percentages and tested with a Chi-square test. The other time intervals were presented with median and IQR, the 90^th^ percentile and the minimum and maximum value and tested with a Wilcoxon rank-sum test.

We used a matched cohort analysis to eliminate possible confounding by differences in diagnosis, age and sex. As mentioned before, we aimed for two matched control patients for every patient at the DC (1:2 ratio). To account for the correlation within matched pairs, we used generalized estimation equations (GEE), which is a nonparametric statistical method that estimates population-averaged effects assuming different correlation structures. We used the difference in number of days between the DC and the control group as outcome and assumed equal correlation within pairs. Because of the highly skewed distribution of time intervals in both groups, we also dichotomized the time intervals into “long” and “short” and estimated the risk ratio (risk for long time intervals among the control patients compared to the DC-patients) by a matched pair-cohort analysis using the Mantel-Haenszel method. The cutoff-values were set to >4 weeks for the primary care interval, >8 weeks for the diagnostic interval, >1 week for the information interval, and >2 weeks for the treatment interval. These cutoff-values were chosen by inspecting the distribution of the time intervals and deemed to be clinically relevant. In this analysis, we only used one matched control per patient at the DC (1:1 ratio; the pair with the best match according to the ranking scheme was chosen). We also presented the different time intervals (except for the patient interval, which was collected as categorical data) in quantile plots to compare the distribution between the DC and the control group.

To compensate for differences between the groups, four sensitivity analyses were performed. In the first, we matched patients on similar symptoms instead of cancer diagnoses. Out of the in total 35 different symptoms, we matched on number of identical symptoms. Matched patients had at the most four identical symptoms and at the least one identical symptom. Age and sex were also included as matching variables. In a second analysis, we excluded patients with focal alarm symptoms. In a third analysis, we excluded pairs where matching on exact cancer diagnosis was not possible and in a fourth set of analyses, we compared number of working days instead of calendar days. In an additional sensitivity analysis, we matched patients with both the same cancer diagnosis and similar symptoms. Due to few possible matches, the results were not analyzed for statistical significance, but are presented in the text. All statistical analyses were done in STATA version 15 (StataCorp LP).

## Results

### Patient Characteristics

A total of 64 patients were diagnosed with cancer at the DC during the study period (corresponding to 22.1% of all patients that were referred to the DC and gave their consent to take part in the evaluation). Eight of them were excluded since no eligible matched control patients could be identified based on cancer diagnosis. For 42 of the remaining 56 patients, two control patients were identified per each patient and for 14 DC-patients, only one control patient per each patient could be found thus rendering a total of 98 matched pairs. The patients in the control group were contacted after cancer diagnosis and the median time between cancer diagnosis and consenting to participating in the study was 10 months, ranging from 4 to 20 months. The patients at the DC were, in most cases, enrolled in the study before cancer diagnosis (median time from informed consent to cancer diagnosis was 10 days, ranging from 3 months before to 9 days after cancer diagnosis).

Patient characteristics, cancer forms and frequency of nonspecific and focal alarm symptoms for all matched study participants in the two groups are presented in **Table 1**. At the DC, the median age was 71 years (IQR 63–76) and 22 participants (39%) were women. In the control group, the median age was 72 years (IQR 62–77) and 37 participants (38%) were women. The most common cancer form at the DC was hematologic cancers (29%), whereas in the control group colorectal cancers were most common (31%). The five nonspecific symptoms that were the inclusion criteria for referral to the DC were generally more common among the patients at the DC than in the control group. Fewer patients in the DC-group had focal alarm symptoms as compared to the control group (21% vs. 33%). The reason for occasionally including patients with focal alarm symptoms at the DC was that the center was new and they wanted to be inclusive rather than exclusive, when deemed appropriate by the physician that assessed the referrals. The 3-year survival rates at the DC and in the control group were 0.34 (95% CI 0.22–0.47) and 0.81 (95% CI 0.71–0.87) respectively (**Table 1**). When taking comorbidity into account, the difference in survival decreased slightly, but not entirely (data not shown). The complete diagnostic spectrum of the in total 290 DC-patients that consented to take part in the evaluation of the DC has been described elsewhere. At the DC, cancer diagnosis was ruled out when a non-malignant disease could explain the patient’s symptoms (64.1% of all patients) or when no disease could be found (13.8% of the cases, in which group the symptoms had often disappeared during the investigational process) (3).

**Table 1:** Patient characteristics at the DC and in Helsingborg.

	DC (n = 56)	HBG (n = 98)
Age, median (IQR)	71 (63-76)	72 (62-77)
*Sex, number (%)*		
Men	34 (61)	61 (62)
Women	22 (39)	37 (38)
*Cancer diagnoses*, *number (%)*		
Hematologic	16 (29)	19 (19)
Lung	13 (23)	17 (17)
Colorectal	7 (13)	30 (31)
Metastases	7 (13)	0 (0)
Bladder/kidney	7 (13)	10 (10)
Liver/pancreatic	4 (7)	1 (1)
Cancer of unknown primary	3 (5)	3 (3)
Prostate	2 (4)	10 (10)
Miscellaneous	2 (4)	0 (0)
Female reproductive system	2 (4)	6 (6)
Breast	2 (4)	3 (3)
Connective tissues	1 (2)	0 (0)
*Nonspecific symptoms, number (%)*		
Fatigue	17 (30)	20 (20)
Weight loss	28 (50)	15 (15)
Pain/joint pain	22 (39)	20 (20)
Prolonged fever	2 (4)	1 (1)
Abnormal test results	36 (64)	21 (21)
Suspected metastasis	10 (18)	1 (1)
*Focal alarm symptoms, number (%)*	12 (21)	32 (33)
*Previous diagnoses, number (%)*		
Previous cancer	17 (30)	16 (16)
Cardiovascular disease	16 (29)	18 (18)
Diabetes	13 (23)	11 (11)
Chronic obstructive pulmonary disease	5 (9)	3 (3)
*3-year survival rate (95% CI)*	0.34 (0.22–0.47)	0.81 (0.71–0.87)

At the DC, 27 (48%) patients had infiltrating tumors and among the matched controls, 40 (41%) patients had infiltrating solid tumors. 30% of the patients at the DC and 16% of the control patients had a previous cancer diagnosis, 29% of the DC-patients and 18% of the control patients had a history of cardiovascular disease, 23% and 11% respectively were diagnosed with diabetes, 9% and 3% respectively were diagnosed with chronic obstructive pulmonary disease (**Table 1**).

### Time Intervals

The five different time intervals for the DC and the control group are presented in **Table 2**. There was no significant difference in how long the patients waited with seeking care (patient interval) between the DC and the control group. Likewise, there were no significant differences in the median primary care interval (17 days, IQR 5–59 and 16 days, IQR 0–45 respectively) or diagnostic interval (45 days, IQR 24–94 and 38 days, IQR 25–81 respectively) between the DC and the control group. There was a tendency towards shorter median treatment interval at the DC compared to the control group (21 days, IQR 6–33 and 31 days, IQR 16–50 respectively), with a significant difference between the groups in the matched analysis (difference 13 days, 95% CI 0.1–25, p = 0.049; **Table 3**). Included in this interval was the time between cancer diagnosis noted in the medical records and information to the patient about the diagnosis (information interval). This interval was significantly shorter at the DC compared to the control group (1 day, IQR 0–3 and 3 days, IQR 0–12 respectively; **Tables 2** and **3**) and the risk of having to wait a while (more than 1 week) for information about a cancer diagnosis was significantly higher among the control patients (risk ratio 5.0, 95% CI 1.6-16.1; **Table 3**).

**Table 2:** A comparison of time intervals (calendar days) between the DC and Helsingborg.

	DC	HBG	p-Value
*Time interval*			
Patient interval^a^			
n	41	92	0.28
0–3 months	24 (59)	63 (68)	
4–6 months	13 (33)	17 (18)	
7–9 months	2 (5)	2 (2)	
10-12 months	0 (0)	3 (3)	
>1 year	2 (5)	7 (8)	
Primary care interval (days)^b^			
n	56	90	0.57
Median (IQR)	17 (5–59)	16 (0–45)	
p90^f^	111	97	
Min–max	0–210	0–429	
Diagnostic interval (days)^c^			
n	56	92	0.69
Median (IQR)	45 (24–94)	38 (25–81)	
p90^f^	122	128	
Min–max	4–232	0–576	
Information interval (days)^d^			
n	45	86	0.0005
Median (IQR)	1 (0–3)	3 (0–12)	
p90^f^	7	21	
Min–max	0–19	0–115	
Treatment interval (days)^e^			
n	42	82	0.22
Median (IQR)	21 (6–33)	31 (16–50)	
p90^f^	48	71	
Min–max	0–118	0–225	

a Time from first symptom to contact.

b Time from first visit in primary care to referral for the DC/secondary care or diagnosis.

c Time from first visit in primary care to cancer diagnosis.

d Time from cancer diagnosis to patient informed of diagnosis.

e Time from cancer diagnosis to start of treatment.

f 90th percentile (90% of the patients have an investigational interval time below this value).

**Table 3:** Matched analysis of time intervals (calendar days) between DC and Helsingborg.

	Number of obs.	Difference^e^ (HBG-DC)	p-Value	95% CI
Outcome:				
*Difference in time intervals (days) between HBG and DC*				
Primary care interval^a^	90	3	0.77	-18; 24
Diagnostic interval^b^	92	8	0.48	-14; 31
Information interval^c^	70	7	0.001	3; 11
Treatment interval^d^	63	13	0.049	0.1; 25
		Risk ratio^f^ (HBG vs. DC)		
*Risk for long time interval in HBG compared to DC*				
Primary care interval >4 weeks	51	1.1	0.65	0.7; 1.6
Diagnostic interval >8 weeks	52	0.9	0.44	0.6; 1.2
Information interval >1 week	40	5.0	0.007	1.6; 16.1
Treatment interval >2 weeks	37	1.1	0.44	0.8; 1.5

a Time from first visit in primary care to referral for the DC/secondary care or diagnosis.

b Time from first visit in primary care to cancer diagnosis.

c Time from cancer diagnosis to patient informed of diagnosis.

d Time from cancer diagnosis to start of treatment.

e Number of days in Helsingborg—number of days at DC.

f Risk for long time interval in Helsingborg compared to DC.

**Figure 2:**
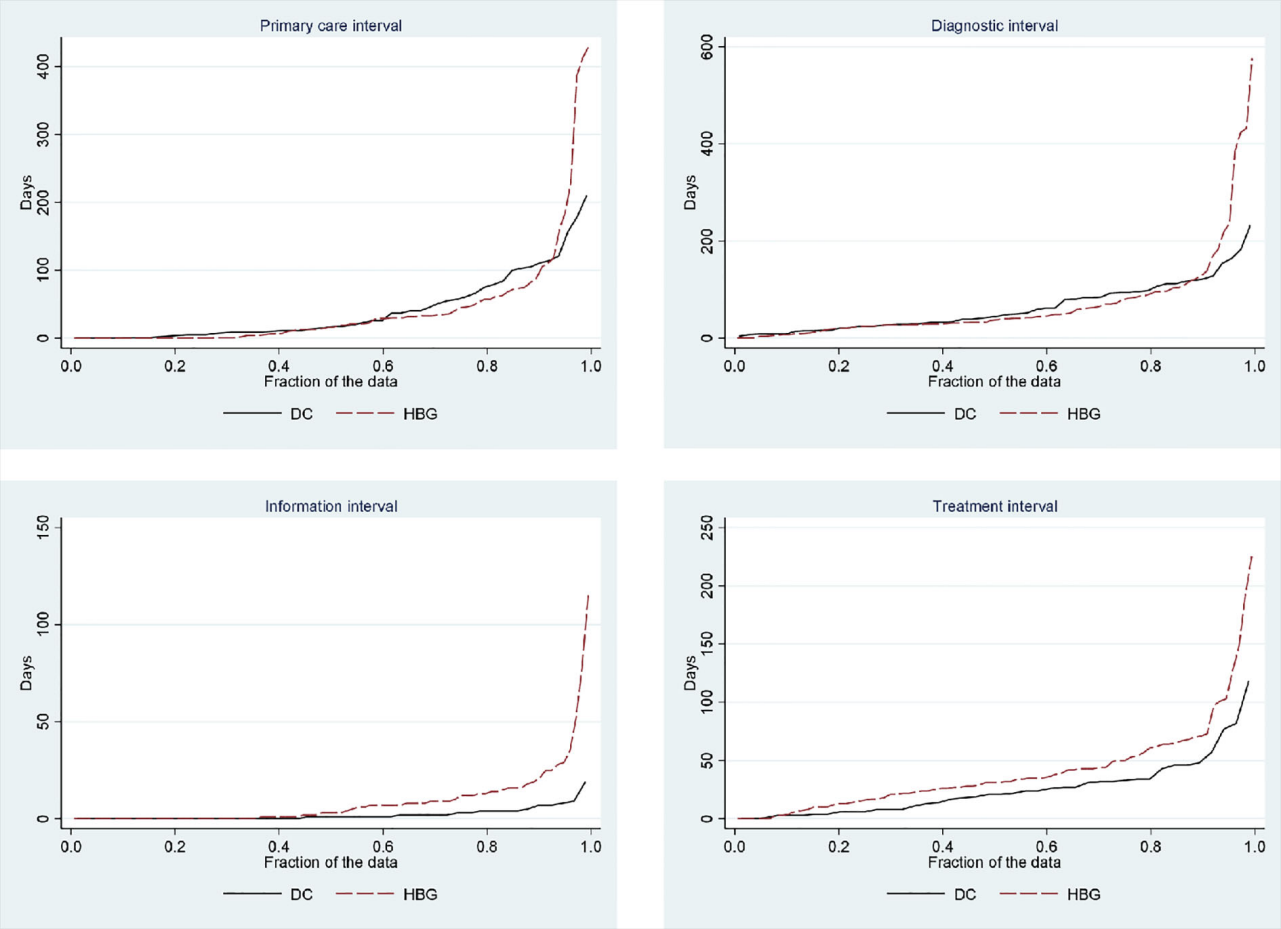
Quantile plots of the time intervals at the DC and in Helsingborg.

**Figure 2** shows the distribution of days for the different time intervals in each group (except for the patient interval where we only had categorical data). The graphs illustrate generally skewed profiles in both groups with most patients having short intervals, but with a few patients drawing towards extreme time intervals. The distributions of the primary care interval and the diagnostic interval were quite similar between the groups for most patients, but a few patients in Helsingborg had much longer intervals compared to the patients at the DC. The information and treatment intervals were slightly shorter at the DC and with a higher share of extreme values in Helsingborg. Overall, it seemed as if extremely long time intervals were avoided at the DC.

The findings in **Table 3** largely remained the same in the additional sensitivity analyses. When matching on similar symptoms instead of cancer forms, there was no difference between the DC and the control group in length of primary care interval or diagnostic interval, but there was still a significantly shorter information interval at the DC (difference 6 days, p = 0.006) and there was also a significantly shorter treatment interval at the DC (difference 27 days, p = 0.001). In this analysis, there were occasional matched pairs in which one individual had an alarm symptom (n = 20; 32% of matched pairs). However, similar results were seen when excluding patients with focal alarm symptoms, when excluding patients that were not possible to match on exact cancer diagnosis, or when comparing working days instead of calendar days. In all these cases, both information and treatment intervals were significantly shorter at the DC (**Supplementary Tables S1–S4**). In an additional sensitivity analysis, we matched on both the same cancer diagnosis and similar symptoms. Only six such matches could be found, but for these, primary care-, diagnostic-, information-, and treatment intervals were all shorter at the DC compared to Helsingborg hospital (time differences: 5 days, 4 days, 7 days and 14 days respectively; data not shown).

## Discussion

In this attempt to put diagnostic time intervals at the DC into context, we could not detect any evidence of shorter diagnostic intervals on a group level compared to another emergency hospital within the same organization. However, extremely long time intervals seemed to be avoided to a higher extent at the DC. The patients at the DC were also informed about their cancer diagnoses earlier and there was a tendency towards shorter time to treatment at the DC compared to the control group. This suggests that the communication around, and with, the patients may benefit from a more comprehensive setup by focusing on patient-centered care and with close collaboration with other clinics.

We were a bit surprised not to find any evidence of shorter total diagnostic intervals at the DC compared to the control group, since reduced time to cancer diagnosis was one of the project’s goals. It may partly be explained by the fact that the DC-model did not shorten the primary care interval as much as expected; in the present study, there were no significant differences in the primary care intervals between the groups. When introducing the DC-model, the goal was a primary care interval that should not exceed 15 days, but in a previous study, we found that only 41% of the patients diagnosed at the DC fulfilled the time goal in primary care (for patients diagnosed with cancer a slightly higher share, 45%) and only 28% had gone through all recommended investigations prior to DC-referral (3). Since the primary care intervals were, in some cases, equal to the diagnostic intervals in the control group, total diagnostic interval was the outcome that was best suited to detect possible diagnostic time differences between the groups, but perhaps the diagnostic intervals can be shortened in the future by improved compliance to the DC-model in primary care.

Despite a thorough matching process, differences between the groups were observed that may have affected the results. Among the DC-patients, hematologic cancers were most common, whereas colorectal tumors were the most common cancer type among the control patients. However, the results remained the same when excluding pairs that did not match on exact cancer diagnosis, thus adjusting for differences in diagnostic spectrum. The patients at the DC had markedly lower 3-year survival rate compared to the control group. Previous studies have actually suggested a higher mortality with very long as well as very short diagnostic intervals, though the latter association, the so called “waiting time paradox”, is believed to be due to unmeasured confounding, e.g., tumor aggressiveness (10, 16, 22). One study suggested that this phenomenon was confined to patients with alarm symptoms. For patients with vague symptoms only, there was no such association (16).

The patients at the DC also had a slightly higher share of infiltrating tumors, more nonspecific symptoms and more comorbidities in their medical history compared to the control group. This is partly in accordance with a previous study, which found that patients with vague symptoms had more advanced stage tumors. However, contrary to our findings, they also had lower comorbidity, whereas the DC-patients had more reported comorbidity compared to controls (16). The reported comorbidity could explain some, but not all, of the differences in survival between the two groups in our study. Thus, the patients at the DC seem to, in general, have been in a worse condition compared to the control group. Since the DC-investigation resulted in a wide range of cancer types, including hematological cancers, it was not possible to control for tumor stage. Regarding comorbidity, we had only access to ICD-codes and not severity of the diseases, thus we chose not to control for this either. The sensitivity analysis matching on symptoms may to some extent compensate for this limitation.

Another difference between the groups was that fewer patients at the DC had focal alarm symptoms compared to the control group [still, the share of patients with alarm symptoms in the control group (33%) was lower than previously reported in a study of all cancers diagnosed after an initial investigation in primary care (49%) (23)]. Instead, the patients at the DC tended to have more of the predefined nonspecific symptoms that were inclusion criteria for referral to the DC. Such vague symptoms may imply a more complicated and lengthy diagnostic workup compared to alarm symptoms where the diagnostic workup is usually straight forward. In semi-structured interviews with general practitioners, the difficulty of detecting cancer in patients with vague symptoms was emphasized (24) and a Danish study examining five common cancers showed that patients with vague symptoms had much longer diagnostic intervals than patients with alarm symptoms (16). Thus, the discrepancy in symptom characteristics in our study could have affected the length of the diagnostic intervals in favor of the control group. However, the findings remained the same when matching on symptoms or excluding patients with alarm symptoms.

Finally, the different recruiting methods used, in which the DC’s patients were consecutively included at first visit whereas the control patients were matched on cancer diagnosis, age and sex, implied that the patients in the control group, in addition to the matching criteria, were selected on survival (since informed consent was a prerequisite for data collection). The DC-patients were all alive when entering the study as well, but they were usually included before the cancer diagnosis. Hence, there was a slight time offset in the control group, which may have introduced a recall bias regarding time for first symptom and subsequently the length of the patient interval. The difference in patient interval was, however, not significant between the groups.

It is possible that the nonspecific symptoms, which were more common in the DC-group, took a longer time for the patients to recognize as signs of disease compared to classical alarm symptoms, as they often occur as normal health variations (perhaps even more difficult to recognize with poor general health). This may explain the higher share of infiltrating tumors at the DC compared to the control group, which in turn may be associated with the lower 3-year survival rate. A recent study of presenting symptoms in primary care showed that patients with nonspecific symptoms were more likely to be diagnosed at a late cancer stage compared to patients with focal alarm symptoms (25). A semi-structured qualitative interview study found that the participants attributed nonspecific cancer symptoms to co-morbidities or other benign causes such as menopause and that these issues could cause long patient intervals (26). There are, to the authors’ knowledge, no easy solutions to the problem of recognizing nonspecific symptoms as signs of cancer, considering the risk of unnecessary investigations and over-diagnosis. One clue may be increased consultation frequency in primary care, which has been shown to occur the year before cancer diagnosis (27). However, nonspecific symptoms are not always related to late cancer stages. This has been demonstrated in a recent study of 12 solid tumor types, which showed that more than half of the patients with weight loss, fatigue or abdominal pain had TNM stages below stage IV (28). Urgent referral pathways for patients with nonspecific symptoms are therefore motivated. It should also be noted that the concept of the DC was not only detection of cancer at an earlier stage, but also to offer patients with nonspecific symptoms a short diagnostic workup with a comprehensive set of samples and investigations aimed at preventing unnecessary referrals between clinics and further delay of diagnosis and treatment, including palliative care. Our results suggest that the DC-project achieved this to some extent since extreme diagnostic intervals were rare, possibly because of a standardized diagnostic workup with efficient communication around and with the patient.

The Danish cancer plan for patients with nonspecific or vague symptoms has a slightly different setup than the Swedish DC. They use a three-legged strategy, with urgent referral for alarm symptoms, the “nonspecific symptoms and signs of cancer patient pathway” (NSSC-CPP, equivalent to the Swedish DC) and No-yes clinics for patients with “low-risk-but-not-no-risk” symptoms, where the general practitioner keeps the responsibility for the diagnostic workup with direct access to fast investigations (2). In Sweden, the latter patient group is investigated in primary care unless there is a clear suspicion of cancer; then, they may be eligible for referral to the DC. The diagnostic workup is rather similar between the Danish NSSC-CPP and the Swedish DC, with initial blood tests and medical imaging in primary care followed by investigations at a diagnostic unit (3, 18). However, the NSSC-CPP has more alternatives for finalizing the investigational course, including “cancer no longer suspected” and “still strong suspicion of cancer with referral to an organ-specific pathway” (17). At the Swedish DC, the investigations usually continue until a specific diagnosis can be identified or excluded. Subsequently, the median investigational duration has been shorter at the Danish diagnostic units: 7 days, compared to 11 days at the Swedish DC (3, 18). The Norwegian pathway for nonspecific, serious symptoms is similar to the Danish and Swedish models, but without standardized medical imaging in the first phase (4). The English Suspected CANcer (SCAN) pathway also has a similar arrangement, but the patients should be ≥40 years (5), which may be worth considering in Sweden as well since cancer before that age is uncommon (29).

The matched control group design used in the present project, including patients from another hospital within the same county, was the best available alternative considering our conditions with lack of historical data and no possibility to randomize patients into different study arms, but as discussed, the design has inevitable limitations. Nevertheless, both hospitals in the study belong to the same healthcare organization with similar guidelines and routines. Both are emergency hospitals of similar size and the patients were recruited during the same time period and with the first part of the investigation in primary care. The sensitivity analyses also strengthen the results.

It should be noted that the statistical power was calculated based on the expected number of patients needed for proper analyses of the DC’s test packages and not time differences, thus the sample size was small and the number of matched pairs limited. Further analyses of similar fast-track referral pathways for patients with nonspecific symptoms are needed to draw reliable conclusions about the effect on different diagnostic time intervals. The findings from our study should be interpreted with this limitation in mind. Still, our results suggest the hypothesis that the primary gain of a DC-model may not be shorter diagnostic intervals, but instead faster handling after detection of cancer along with high patient satisfaction as shown before (3). Whether the differences in median information and treatment intervals were clinically significant can be discussed, but the distributions differed between the groups with some very long intervals in the control group.

## Conclusions

This study showed no significant differences in patient interval, primary care interval or total diagnostic interval on a group level between patients diagnosed with cancer at the DC and the matched control group at Helsingborg hospital. On the other hand, extremely long time intervals seemed to be avoided to a higher extent at the DC, patients were informed earlier about their cancer diagnoses and there was a tendency towards shorter treatment interval compared to the control group, with a significant difference in the matched analysis and sensitivity analyses.

Due to the limitations regarding comparability between the groups as described above, the results must be interpreted with caution and should preferably be considered as hypothesis generating in the design of future studies with a possibility of e.g. randomization. Even so, the results suggest that there might be room for improvement of the DC-model by having further efforts on implementation in primary care (this work has started *via* a new Swedish standardized pathway recommending a primary care investigation of maximum 5 days). It is possible that similar models in other countries face slightly different challenges, e.g. depending on whether the family physicians have gatekeeping-roles or not (30). However, implementation of new evidence in healthcare is a global challenge and further research about this is warranted (31). Already now, patients that are diagnosed with cancer at the DC may benefit from the early information and timely management in the form of active treatment or palliative care.

Our findings provide a small piece of the puzzle in the currently ongoing extensive research about fast track referral in cancer diagnostics. Future evaluations of the different test packages and health economic aspects of the DC-model could be useful to further refine the concept.

## Data Availability Statement

The raw data supporting the conclusions of this article will be made available by the authors, without undue reservation.

## Ethics Statement

The studies involving human participants were reviewed and approved by the Regional Ethics Committee, Lund, Sweden. The patients/participants provided their written informed consent to participate in this study.

## Author Contributions

JS, SR, CS, JJ, and ES contributed to planning and carrying through the project and manuscript preparation. KP performed the data analyses and contributed to the manuscript preparation. All authors contributed to the article and approved the submitted version.

## Funding

The project was funded by the Regional Cancer Centre South, Region Skåne, Sweden, and the Center for Primary Health Care Research, Region Skåne/Lund University, Sweden.

## Conflict of Interest

The authors declare that the research was conducted in the absence of any commercial or financial relationships that could be construed as a potential conflict of interest.
