# Supplementary material for: Time Intervals Under the Lens at Sweden’s First Diagnostic Center for Primary Care Patients With Nonspecific Symptoms of Cancer. A Comparison With Matched Control Patients
Source: Front Oncol. 2020 Nov 30;10:561379. doi: 10.3389/fonc.2020.561379 (PMC7735559; doi:10.3389/fonc.2020.561379)
Supplement: Supplementary file 2 [file Table_2.docx]

Table S2. No focal alarm symptoms: Matched analysis of time intervals (calendar days) between DC and Helsingborg.

|  | Number of obs. | Difference^e^ (HBG-DC) | p-value | 95% CI |
| --- | --- | --- | --- | --- |
| Outcome: |  |  |  |  |
| *Difference in time intervals (days) between HBG and DC* |  |  |  |  |
| Primary care interval^a^ | 47 | 14 | 0.33 | -14; 42 |
| Diagnostic interval^b^ | 49 | 23 | 0.20 | -12; 57 |
| Information interval^c^ | 37 | 8 | 0.03 | 0.6; 15 |
| Treatment interval^d^ | 33 | 18 | 0.06 | -0.5; 37 |

^a^Time from first visit to referral for the DC/secondary care or diagnosis

^b^Time from first visit to cancer diagnosis

^c^Time from cancer diagnosis to patient informed of diagnosis

^d^Time from cancer diagnosis to start of treatment

^e^Number of days in Helsingborg – number of days at DC
