# Supplementary material for: Time Intervals Under the Lens at Sweden’s First Diagnostic Center for Primary Care Patients With Nonspecific Symptoms of Cancer. A Comparison With Matched Control Patients
Source: Front Oncol. 2020 Nov 30;10:561379. doi: 10.3389/fonc.2020.561379 (PMC7735559; doi:10.3389/fonc.2020.561379)
Supplement: Supplementary file 3 [file Table_3.docx]

Table S3. Pairs with no match on exact cancer diagnosis dropped: Matched analysis of time intervals (calendar days) between DC and Helsingborg.

|  | Number of obs. | Difference^e^ (HBG-DC) | p-value | 95% CI |
| --- | --- | --- | --- | --- |
| Outcome: |  |  |  |  |
| *Difference in time intervals (days) between HBG and DC* |  |  |  |  |
| Primary care interval^a^ | 53 | 0 | 1.00 | -24; 24 |
| Diagnostic interval^b^ | 54 | 4 | 0.76 | -20; 27 |
| Information interval^c^ | 41 | 9 | 0.002 | 3; 15 |
| Treatment interval^d^ | 40 | 15 | 0.03 | 1; 29 |

^a^Time from first visit to referral for the DC/secondary care or diagnosis

^b^Time from first visit to cancer diagnosis

^c^Time from cancer diagnosis to patient informed of diagnosis

^d^Time from cancer diagnosis to start of treatment

^e^Number of days in Helsingborg – number of days at DC
