# Supplementary material for: Time Intervals Under the Lens at Sweden’s First Diagnostic Center for Primary Care Patients With Nonspecific Symptoms of Cancer. A Comparison With Matched Control Patients
Source: Front Oncol. 2020 Nov 30;10:561379. doi: 10.3389/fonc.2020.561379 (PMC7735559; doi:10.3389/fonc.2020.561379)
Supplement: Supplementary file 4 [file Table_4.docx]

Table S4. Working days instead of calendar days: Matched analysis of time intervals (working days) between DC and Helsingborg.

|  | Number of obs. | Difference^e^ (HBG-DC) | p-value | 95% CI |
| --- | --- | --- | --- | --- |
| Outcome: |  |  |  |  |
| *Difference in time intervals (days) between HBG and DC* |  |  |  |  |
| Primary care interval^a^ | 90 | 3 | 0.72 | -11; 17 |
| Diagnostic interval^b^ | 92 | 5 | 0.48 | -10; 20 |
| Information interval^c^ | 70 | 4 | 0.003 | 2; 7 |
| Treatment interval^d^ | 63 | 8 | 0.03 | 1; 16 |

^a^Time from first visit to referral for the DC/secondary care or diagnosis

^b^Time from first visit to cancer diagnosis

^c^Time from cancer diagnosis to patient informed of diagnosis

^d^Time from cancer diagnosis to start of treatment

^e^Number of days in Helsingborg – number of days at DC
